# Volunteer-supported Care Transition Interventions for People Living with Dementia: A Secondary Analysis of a Scoping Review

**DOI:** 10.5334/ijic.9056

**Published:** 2025-05-21

**Authors:** Sidra Bharmal, Michelle Nelson, Marianne Saragosa

**Affiliations:** 1 Lunenfeld-Tanenbaum Research Institute, Sinai Health, Toronto, Ontario, Canada; 2 Science of Care Institute, Sinai Health, Toronto, Ontario, Canada; 3 Institute of Health Policy, Management and Evaluation, University of Toronto, Toronto, Ontario, Canada

**Keywords:** dementia, unmet needs, volunteer, transitional care, community, older adults

## Abstract

**Introduction:**

Rising dementia rates can worsen the strain on the healthcare system and increase hospital admissions. Hospitals decondition persons living with dementia (PLWD), for which volunteers can offer support. We reviewed existing literature on volunteer-led/supported care transition services available to PLWD, assessing PLWD representation and the extent to which their needs are addressed.

**Methods:**

We conducted a secondary analysis of a scoping review examining volunteer and third-sector personnel providing post-discharge support. Of the review’s 49 articles, we considered services offered to PLWD and persons with cognitive impairment (PWCI). The Camberwell Assessment of Needs for the Elderly (CANE) guided the thematic analysis.

**Results:**

Four of our nine selected articles highlighted services supporting PLWD, though only one was developed explicitly for them. The most common themes of needs targeted or met were physical health (n = 7), company (n = 7), food (n = 6), medications (n = 6), and psychological distress (n = 6).

**Discussion:**

We described the characteristics and outcomes of these volunteer-led/supported care transition interventions. Comparing the leading PLWD needs against those the interventions primarily addressed revealed potential oversight of their most critical needs. However, volunteers remain valuable in supporting discharged community-dwelling PLWD.

**Conclusion:**

In hospital-to-home care transitions, volunteer-led/supported transitional care models benefit PLWD and their caregivers. However, few available interventions explicitly focus on this patient population. Therefore, this is an opportunity to understand better how volunteers and third-sector organizations could optimally support those living during care transitions through an integrated care approach.

## Introduction

With a rapidly growing population of older adults worldwide [1], the incidence of dementia is following a similar trend [1,2]. Dementia, one of the most common neurodegenerative diseases, is projected to rise in prevalence from 50 million cases in 2018 to an estimated 150 million in 2050 [2,3]. Living with dementia is associated with overall functional disability for older adults, and the lack of services suited to manage such difficulties may impose some challenges on those affected and their support networks, including families and healthcare systems [2,3].

Persons living with dementia (PLWD) have many complex care needs, which, when unmet, can negatively impact their quality of life [4,5]. For example, everyday unmet needs fall within the domains of emotional and psychological (e.g. distress), social (e.g. loneliness), physical (e.g. vision or hearing impairment), self-care and sanitation (e.g. bathing), and financial [6,7,8,9]. Most of the population of PLWD age in place (70%), which contributes to different types of concerns related to the accessibility of the healthcare system, safety, daily activities and structure, information, special aids, dementia-related constraints and acceptance, relationships, and food [6,9]. These complex care needs lead PLWD to access healthcare services more frequently, putting pressure on the healthcare system [2,8].

As more frequent health system users, hospitalizations are unfortunately common in healthcare trajectories for PLWD [10]. Notably, PLWD experience increased hospital admission rates and longer hospital stays, being especially common for PLWD with other conditions or comorbidities [11,12]. With restricted mobility and social interaction, hospitals are not conducive environments for PLWD and can put them at risk for deconditioning, worsening their health and well-being [10,13]. Therefore, hospitalized PLWD experience exacerbated care needs upon discharge home [14,15].

PLWD have more unsuccessful transitions from hospital to home compared to those without dementia, with higher readmission and mortality rates [16]. Caregivers often find this transition challenging, reporting a need for more readiness to cope with and manage the needs of PLWD [15,17]. Additional support and resources are necessary to help make these transitions out of the healthcare system smoother [14,18,19,20]. Integrated care emphasizes improving these transitions by providing accessible and quality community-based services and support for PLWD and their care partners. While substantial evidence of transitional care models supporting older adults is available [21], attention is starting to be paid to intersectoral approaches to aftercare delivery [22]. Nevertheless, these integrated care practices rely on formal multidisciplinary healthcare teams [23,24]. Emerging integrated care delivery opportunities are found in the closer integration of health and the volunteer sector [25,26].

Volunteers are critical in promoting and delivering community health initiatives [27]. While considered a low-cost and underutilized resource, volunteers help address patients’ unmet health and social needs [28,29]. Evidence shows that volunteer-led/supported care transitions have positively impacted the medically ill and older adult populations [30,31], leading to emotional well-being, lifestyle improvements, and declines in hospital visits and social isolation [32,33]. Therefore, by utilizing volunteer-engaged transitional care programs, community-based organizations can implement sustainable, high-quality, and cost-effective methods that capitalize on the expertise and skills of trained volunteers [28].

This manuscript presents a secondary analysis of the results of a scoping review on existing volunteer-led/supported transitional care models. The objective of the initial scoping review was to explore the nature of the engagement of volunteers and partnerships with third-sector organizations during care transitions, explicitly focusing on the setting where the transitional models were implemented, the program and client characteristics, and the relevant knowledge gaps [34]. While this paper demonstrates the existing literature that describes volunteers’ valuable role in facilitating transitions from hospital to home, we need to find out how these interventions target or benefit PLWD. This has significant implications, presenting an affordable solution that increases accessibility to specialized care and identifying how volunteers can support PLWD best after discharge from the hospital. Therefore, we build on the findings of this scoping review by focusing on volunteer services that include or target PLWD. This secondary analysis asks: How do volunteer-led/supported care transition interventions involve PLWD and address their needs?

## Methods

### Scoping Review Procedures

The primary scoping review followed the methodology outlined by the Joanna Briggs Institute with the PRISMA extension to obtain literature examining the role of volunteers and third-sector organizations in supporting hospital-to-home transitions. Since this particular research area is emerging, a scoping review is useful for providing an overview of the existing literature and mapping the available evidence in knowledge. The eligibility criteria were based on PCC (Population/Concept/Context) characteristics. The population involved adults (≥18 years) receiving a service supporting the transition from hospital to home. The concept of interest was volunteer-supported care transitions. Volunteers were defined as individuals in a formal position with a third-sector organization involved in activities or services without compensation for time or expertise. The context was any service within a third-sector organization. Third-sector organizations were classified as non-government, nonprofit, charitable, faith-based, or social enterprises.

The database search strategy was developed by an Information Scientist for Ageline, CINAHL, Cochrane Reviews, EMBASE, Joanna Briggs (JBI), OVID Medline, PsycInfo, Scopus, Social Work Abstracts, and Sociological Abstracts. Examples of search term combinations included terms and phrases synonymous with “volunteer,” “nonprofit service,” “post-discharge,” “transition,” or “community reintegration” (Supplemental Table 1. provides a complete list of search terms for each database). The grey literature search was conducted through ProQuest, targeted Google searches, and relevant organizations’ websites. Unpublished research, program summaries, evaluation reports, theses and dissertations, organizational reports, conference proceedings, and relevant newspaper articles were identified. Lastly, references for relevant reviews were searched, and experts in transitions were consulted to provide more literature. The evidence included was published from 2000 to 2023.

**Figure 1:**
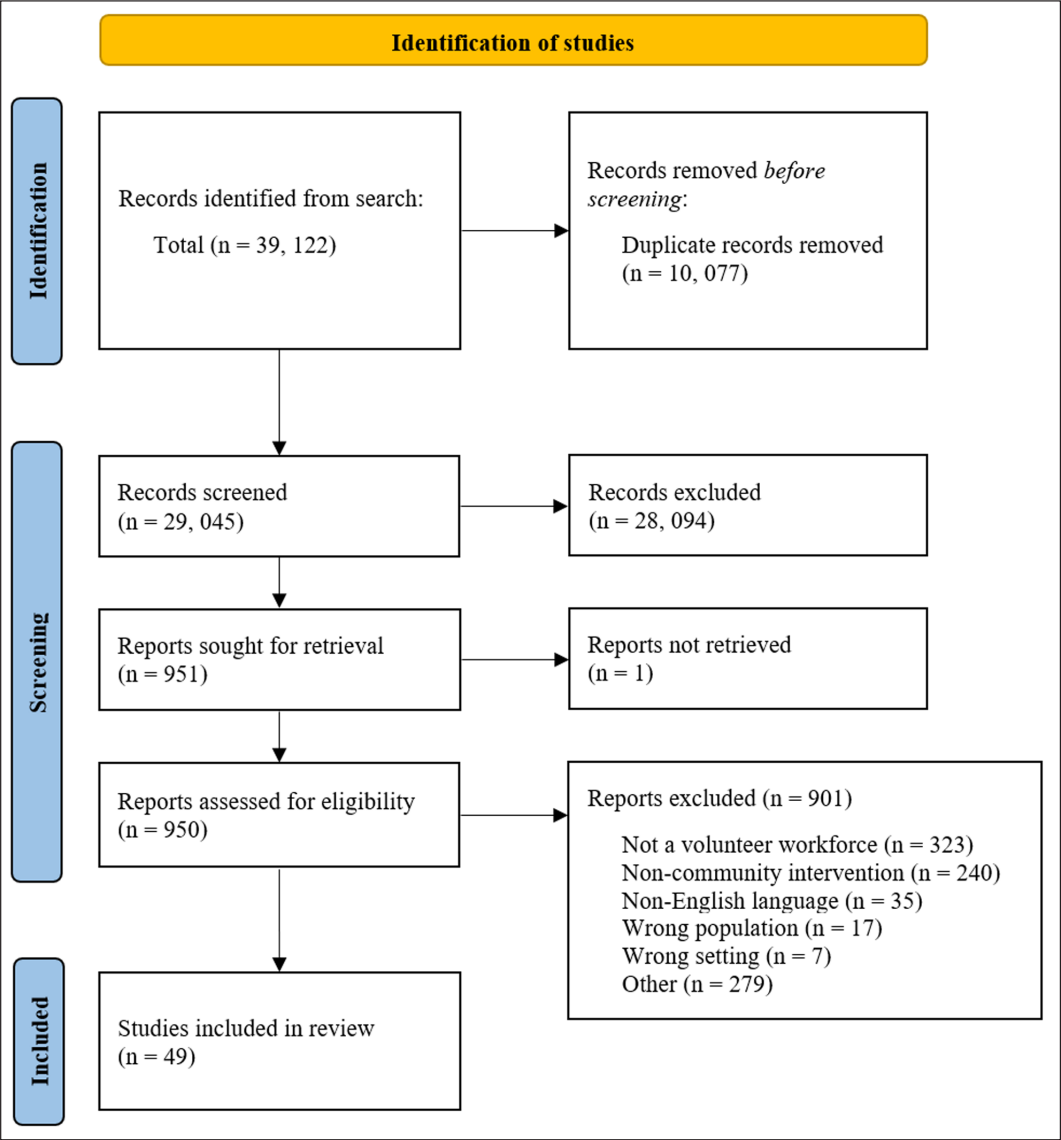
PRISMA flowchart of article screening for scoping review on the role of volunteers and third-sector organizations in supporting transitions from hospital to home.

The search produced 39,122 articles, of which 10,077 were duplicates and were removed. The final scoping review articles were selected through a rigorous 2-step screening process and yielded 950 articles remaining after level one screening. Exclusion criteria for full-text screening included no mention of a volunteer workforce, not a community-based intervention, not published in English, population beyond the review’s focus, or inappropriate setting. 901 articles were excluded after review at the full-text level, resulting in 49 remaining articles [34]. Figure 1 displays the PRISMA flowchart describing article selection.

### Secondary Analysis of Scoping Review Procedures

Secondary analyses describe repurposing and maximizing the data from a previous study to explore a different research question [35]. They are typically applied when investigating new areas distinct from the original analysis, conducting additional analysis of the original data, a subset of the original data, or newly collected data not explored in the original analysis, shifting the focus to examine different facets of the original analysis, or validating or building on findings from the original analysis [36].

**Figure 2:**
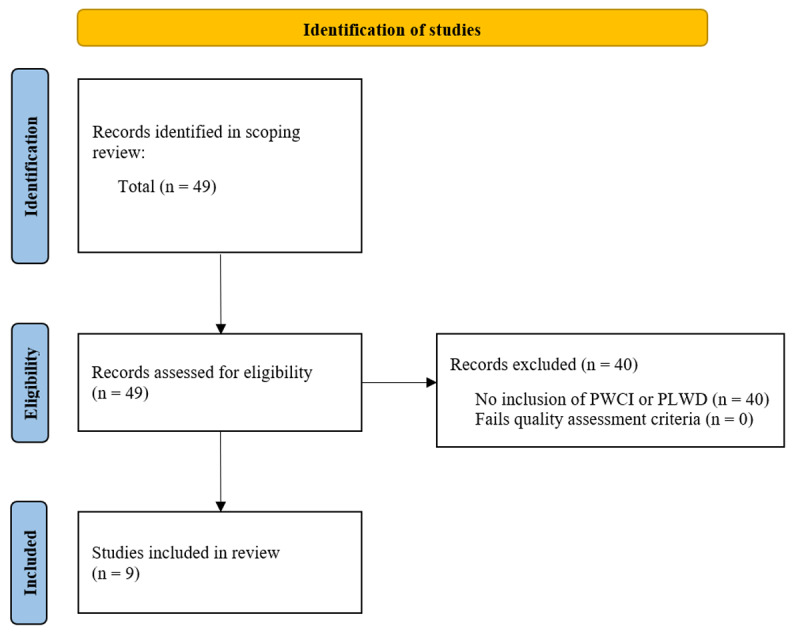
Literature included in a secondary analysis of the scoping review examining the extent and nature of transitional support programs integrating volunteers and third-sector personnel. This analysis included studies involving persons living with dementia (PLWD) and other persons with cognitive impairment (PWCI).

We conducted a secondary analysis of the nine articles in the scoping review that had samples involving PLWD. Given the infrequent mention of PLWD in the studies, we broadened the scope to include persons with cognitive impairment (PWCI). We defined cognitive impairment as difficulty with thinking, memory, learning, decision-making, judgement, or concentrating. Articles that did not report or explicitly excluded PLWD and/or PWCI as recipients of their interventions were excluded from the secondary analysis. We did not filter based on literature or study design type, introducing variability in the depth of reporting. This heterogeneity reflects the broad scope of the review and highlights the diversity in approaches to volunteer-supported care transitions. Article selection is outlined in Figure 2. One screener (SB) selected the articles and conducted data extraction, with the support of the senior author (MS) for difficult to interpret cases. Inclusion or exclusion was determined through discussion and mutual agreement. Each article was reviewed in depth, and the data extracted from the articles selected included the following characteristics: location, study design, target population, average age, diagnosis or nature of impairment, intervention (description, duration, dosage, setting, and provider), volunteer training requirements and curriculum, patient outcome measures, and findings (Supplemental Table 2). All articles from the initial scoping review were appraised for relevance, reliability, validity, and applicability (Supplemental Table 3).

We consulted the Camberwell Assessment of Needs for the Elderly (CANE) tool to provide context on the unmet needs of community-dwelling PLWD. We applied these needs as themes in our thematic analysis. Articles were reviewed to determine if they addressed the established themes, allowing focused extraction and categorization based on the presence of relevant content. The CANE is a validated tool used by many studies to understand the needs of PLWD [37,38,39]. We also considered a systematic review and meta-analysis of studies obtaining data based on CANE that represent the frequency of these needs [40]. Table 1. demonstrates the estimated prevalence of CANE-structured needs reported by PLWD and their caregivers.

**Table 1:** Ranked proportions of PLWD- or caregiver-reported dementia needs itemized by CANE, estimated by Curnow et al. [40] Data is based on 1011 PLWD and 1188 informal caregivers of PLWD from six retrieved studies.


	PLWD-REPORTED NEEDS		CAREGIVER-REPORTED NEEDS	
	
	NEED	PREVALENCE	NEED	PREVALENCE

1	Memory	0.713	Memory	0.933

2	Food	0.706	Household activities	0.886

3	Household activities	0.677	Money	0.855

4	Money	0.566	Food	0.839

5	Physical health	0.526	Daytime activities	0.722

6	Mobility and falls	0.400	Physical health	0.707

7	Daytime activities	0.395	Self-care	0.637

8	Eyesight/hearing	0.380	Medications	0.531

9	Medications	0.371	Mobility and falls	0.511

10	Company	0.324	Psychological distress	0.509

11	Psychological distress	0.293	Company	0.476

12	Self-care	0.283	Eyesight/hearing	0.455

13	Information	0.226	Accidental self-harm	0.318

14	Benefits	0.153	Continence	0.287

15	Continence	0.150	Information	0.256

16	Accommodation	0.128	Benefits	0.183

17	Accidental self-harm	0.109	Accommodation	0.177

18	Intimate relationships	0.108	Psychotic symptoms	0.175

19	Psychotic symptoms	0.047	Behaviour	0.125

20	Caring for another	0.045	Intimate relationships	0.114

21	Deliberate self-harm	0.036	Abuse/neglect	0.063

22	Behaviour	0.024	Alcohol	0.057

23	Abuse/neglect	0.015	Caring for another	0.049

24	Alcohol	0.009	Deliberate self-harm	0.034


### Ethics

As this was a secondary analysis of an existing scoping review, ethical approval was not required, and the primary review also did not seek ethical approval.

## Results

Nine of the 49 articles from the initial scoping review met the inclusion criteria, with four specific to PLWD [41,42,43,44]. Two articles were published by the same author (Eaton) [45,46]); therefore, eight interventions were represented. All articles passed the quality assessment. However, the mixed methods studies did not meet the criterion that required addressing the variations between quantitative and qualitative results [43,46,47], and one protocol paper could not meet any result-related criteria due to the absence of reported results [47].

### Descriptive Characteristics

The included articles were published between 2005 [47] and 2022 [43]. Four locations were represented: United Kingdom (n = 3) [42,43,44], United States (n = 3) [41,47,48], Canada (n = 2) [45,46], and Hong Kong (n = 1) [49]. Only one article was considered grey literature [42] – a guide describing multiple programs assessed through comparison groups and pre- and post-approach. The remaining eight were peer-reviewed [41,43,44,45,46,47,48,49] and could be classified as mixed methods (n = 2) [43,46,47], qualitative (n = 3) [44,45,48], quasi-experimental (n = 1) [41], and RCT (n = 1) [49]. Most articles assessed the impact of the care transition intervention described (n = 6) [41,42,43,44,48,49], while the remaining were a protocol (n = 1) [47], feasibility and acceptability study (n = 1) [46], or training curriculum (n = 1) [45].

### Populations Served

The range of reported mean ages within three articles was 48.8 [46] to 83.1 [43], while the remaining six did not report mean age. Older adults were most often targeted in the samples (n = 5) [41,43,44,48,49], followed by HIV-positive patients (n = 2) [45,46], persons with mental illnesses (n = 1) [47], or lacking specific criteria (n = 1) [42]. There was some overlap related to diagnoses of dementia or cognitive impairment. Non-specific diagnosis of dementia was described in three articles [41,42,43], while two specifically referenced persons with Alzheimer’s [41,44]. Cognitive impairment, in general, was covered in six articles [41,43,44,45,47,49], and an additional article specifically focused on HIV-associated neurocognitive disorder [46].

### Intervention Delivery

Four interventions contacted participants using in-person and remote components [41,42,48,49]; two only had remote components [45,46], and one only had in-person components [44]. Two articles did not report the intervention setting [43,47]. Activities common among the interventions included phone calls (n = 6) [41,42,45,46,48,49], home visits (n = 5) [41,42,44,48,49], discharge planning and assessment (n = 4) [43,47,48,49], goal setting (n = 3) [43,45,46], and social prescribing (n = 3) [43,47,48]. There was significant variation in the frequency of communication with participants, ranging from one [45,46,49] to five [41] weekly interactions or one [44] to 3.5 [41] hours per week. Regarding this contact with participants, four interventions changed in nature or dosage as it progressed [41,45,46,49]; two were flexible depending on other conditions [42,47], two were not specified [43,48], and one remained constant [44]. The duration of volunteer-engaged interventions was described in seven articles [41,43,44,45,46,48,49], ranging from four weeks [49] to seven months [48].

### Volunteer Involvement

Four interventions were primarily carried out by volunteers (i.e. “volunteer-led”) [41,42,44,46], while volunteers were not the leading implementers in five (i.e. “volunteer-supported”) [43,45,47,48,49]. Other health or service providers involved were nurses (n = 4) [43,45,47,49], volunteer coordinators (n = 3) [41,42,43], social workers (n = 3) [43,44,49], general practitioners (n = 2) [43,47], mental health professionals (n = 2) [43,47], counsellors (n = 1) [47], physiotherapists (n = 1) [43], psychologists (n = 1) [47], psychiatrists (n = 1) [47], pharmacists (n = 1) [43], case managers (n = 1) [47], and consumers and family advocates (n = 1) [47].

Volunteer training for the interventions was described in 5 articles [41,42,45,46,49]. The training duration was nine [49] to 44 [45,46] hours. Themes were: safety (e.g. creating safe spaces) (n = 5) [41,42,45,46,49], health promotion (n = 5) [41,42,45,46,49], health conditions (e.g. dementia or pneumonia) (n = 4) [41,42,45,46], communication (n = 4) [41,45,46,49], volunteer role and parameters (n = 3) [42,45,46], anti-oppression and ethics (n = 3) [45,46,49], social resources (n = 2) [41,49], aging aspects (n = 2) [41,49], safeguarding (n = 2) [41,42], confidentiality and disclosure (n = 1) [42], medication and disease self-management (n = 1) [41], referrals for professional help (n = 1) [49], moving and assisting (n = 1) [42], and adult learning principles (n = 1) [41].

### Assessment Measures

Patient experience was reported with quality of life (n = 2) [47,49], satisfaction (n = 2) [48,49], and mood and anxiety (n = 1) [42]. Health outcomes involved symptoms and episodes (n = 2) [43,47], number of falls (n = 1) [42], and nutrition and hydration (n = 1) [42]. Participant functioning involved independence or self-efficacy (n = 3) [47,48,49], social functioning (n = 2) [47,48], and employment functioning (n = 1) [47]. Healthcare burden was evident by hospital admissions or readmissions (n = 5) [41,42,43,48,49], length of hospital stays (n = 2) [42,43], and delay in transfer of care (n = 1) [42]. Other outcomes included meeting needs (n = 2) [47,48], cost of hospital admissions (n = 1) [41], proactive discharge (n = 1) [43], and family burden (n = 1) [47]. Three articles did not report patient outcomes [44,45,46].

In two studies, volunteer-led/supported care transition interventions improved patient experience, mood and anxiety scores [42], and enhanced quality of life and satisfaction with care [49]. Positive health outcomes included reduced length of episodes [43] and better nutrition and hydration [42]. Participant functioning, notably self-efficacy [49], was improved. Decreases in health and social care burden were evident with fewer referrals to or usage of these systems [43] and hospital admissions or readmissions [41,48,49]. However, one study challenged the intervention impacting healthcare demand, determining that it did not affect readmissions, length of hospital stays, delayed transfer of care, and number of falls [42]. In two papers, the interventions successfully tracked individual patient goals, met their perceived needs [45,48], served as their safety net [48], and validated their feelings and experiences [45]. However, the emotional connection can be challenging to maintain over the phone [45,46]. Feasibility and acceptability were confirmed in one study [46].

### Costs

Details on the costs of the intervention components were provided in four articles [41,42,45,46]. According to them, elements to consider when budgeting include honorariums [45,46], staff (e.g. project coordinator) [41,42], material and equipment [41,42], impact measurement costs [42], volunteer recruitment, training, and management [42], and profile raising and visibility [42]. Cost-offset analysis was also explained in three articles [41,42,43]. One acted as a guide for conducting this process, involving total cost, cash outlay, and cost savings during and after a designated time [42]. The other two articles estimated the costs of healthcare services, showing a reduced average cost of ED/hospital visits and that these reduced hospital admissions and early support discharge comprise the most significant cost savings [41,43].

### Needs Met

When evaluated against the CANE tool, papers by McLeod (n = 12) [44], DeForge (n = 11) [47], Dye (n = 10) [41], and Wong (n = 9) [49] met the most patient needs, while Elston [43] met the fewest (n = 2). The most targeted or met needs were physical health (n = 7) [41,43,44,46,47,48,49] and company (n = 7) [42,44,45,46,47,48,49], followed by food (n = 6) [41,42,44,45,47,48], medications (n = 6) [41,45,46,47,48,49], psychological distress (n = 6) [42,43,44,46,47,49], money (n = 5) [41,44,45,47,49], information (n = 5) [41,44,45,47,49], mobility and falls (n = 4) [41,44,47,48], daytime activities (n = 4) [41,42,44,49], behaviour (n = 4) [41,47,48,49], accommodation (n = 3) [46,47,49], memory (n = 2) [41,42], accidental self-harm (n = 2) [41,42], self-care (n = 2) [41,46], intimate relationships [44,46], deliberate self-harm (n = 2) [44,45], household activities (n = 1) [44], eyesight/hearing (n = 1) [44], psychotic symptoms (n = 1) [47], and abuse/neglect (n = 1) [41]. Needs not met by any articles included benefits, continence, caring for another, and alcohol.

## Discussion

This paper reported a secondary analysis of a scoping review of volunteer-engaged care transition interventions. It examined the extent to which PLWDs are included and their needs met. We identified nine articles with PLWD or PWCI as part of the samples, though only one specifically targeted this patient population, indicating little evidence supporting tailored interventions [42]. This represents a gap in the literature presenting *how* volunteer-based initiatives are designed to involve PLWD and PWCI to address their care transition needs effectively. Volunteers in dementia care in hospital settings routinely engage PLWD in one-on-one social activities and even assist with dietary intake [50]. Volunteers are uniquely positioned to provide relational care with some degree of healthcare provider qualities [51].

Instead, the population of interest in the included articles were most often older adults [41,43,44,48,49]. Notably, the remaining 40 articles from the initial scoping review that were not included in this secondary analysis featured three articles where the exclusion of PLWD or PWCI could be confirmed [52,53,54]. An exclusion rationale was provided in one article, stating that the participants must have had adequate cognitive and communication skills to participate in the interviews [52]. Communication is a common challenge for many PLWD, especially as dementia progresses [55]. Therefore, our data suggest that some volunteer-led/supported interventions failed to accommodate PLWD, missing the opportunity to develop or test approaches to overcome their communication barriers and better generalize these interventions to those with the highest risk of hospital readmission [56]. However, as dementia is common among older adults, this could mean that the results are still partially relevant to the subgroup of PLWD and can offer preliminary insights into how these interventions benefit them.

It is worth noting that no evidence was provided regarding the effects of these supports on caregiver outcomes. Without adequate resources and services for PLWD, caregivers often experience excessive burden and psychological distress [57,58]. Neglecting to address their strain undermines the effectiveness of these interventions, as their compromised well-being can significantly impact their ability to facilitate smooth transitions [59,60].

Six of the nine articles (67%) involved remote components in their interventions, relying primarily on phone calls [41,42,45,46,48,49]. This may reflect the adoption of virtual programming with more remotely delivered interventions for older adults being available [61]. Technology adoption and acceptance are rising among older adults [62,63]. Therefore, digital alternatives for activities or services that may be difficult to reproduce or arrange in person could benefit and support this population [63]. Virtual or technological tools can meet needs such as memory, information (including money), physical health, company, and psychological distress, reducing caregiver burden [64]. However, two articles noted that phone call communication can limit emotional connection [45,46]. This emphasizes the importance of a balanced approach in interventions that involve technology, where strategies that maintain a human touch must be incorporated. Four articles with in-person and remote features [41,42,48,49] achieved a comprehensive approach to support.

The intervention durations had a broad range, which brings continuity of care into focus. At the lower end of the spectrum (4 weeks), the intervention reduced 28-day readmissions and improved quality of life, self-efficacy, and satisfaction with care [49]. The intervention at the higher limit (7 months) was linked to reduced 30-day unplanned readmissions with the patient’s individual needs met [48]. While the results are similar regardless of duration, it is essential to consider that the number of readmissions was only calculated while the intervention was in progress. It would be valuable for further studies to examine the effect on readmissions post-intervention or compare different durations to see if the benefits continue. Still, this shows that a volunteer-led/supported intervention can have positive outcomes in such a short timeframe. However, another article reported that their duration (approximately six to eight weeks) was too short as service users could not cope without support and personal contact post-intervention [44]. This demonstrates a need to reduce the participants’ dependency on the service gradually. As the studies progressed, four interventions with positive outcomes attempted this by declining the interactions with the participants [41,45,46,49].

The needs most targeted or met were physical health [41,43,44,46,47,48,49], company [42,44,45,46,47,48,49], food [41,42,44,45,47,48], medications [41,45,46,47,48,49], and psychological distress [42,43,44,46,47,49]. General volunteer-based interventions supporting PLWD in the community have been shown to address many needs, such as memory, food, money, physical health, mobility and falls, daytime activities, company, psychological distress, self-care, information, and intimate relationships [65,66,67,68,69]. Existing literature on volunteer-based interventions for PLWD in hospital settings similarly highlights needs like food, mobility and falls, medications, company, and psychological distress [70]. The consistency across these settings supports the idea that these are pervasive focus areas throughout the continuum of care for PLWD.

While we found that the most frequently addressed needs included physical health, social connection, food, medication, and psychological distress, the most essential needs identified by community-dwelling PLWD are memory, nutrition, household activities, financial support, and physical health [40]. Caregivers of PLWD reported similar significant needs but placed greater emphasis on daytime activities over physical health [40]. The disconnect between the primary needs of PLWD and those most frequently addressed by volunteer-led or supported interventions suggests that these interventions may not be optimally suited for PLWD. However, further research is required to draw definitive conclusions. We have established that nearly all initiatives were not designed for PLWD or PWCI, and we now recognize that their highest priority needs remain unaddressed, even though this demographic is included as recipients. Tailoring these interventions to meet their specific needs is essential to enhance their effectiveness, support, and improve positive outcomes. This is not to imply that the commonly addressed needs are unimportant. As key factors influencing quality of life, a lack of social connection and a decline in psychological well-being can lead to loneliness, depression, anger, and confusion [71]. Moreover, medication management should not be neglected, as difficulties in this area can significantly increase the risk of adverse events for PLWD, potentially leading to hospitalization [72].

Another aspect to consider is that transitioning from hospital to home could lead to a shifted ranking of the critical needs of PLWD. Physical health remains a key indicator of successful care transitions [59]. Since PLWD and caregivers are often not given adequate time to understand the management of their medications, the theme of medication management emerges as a crucial factor [73,74,75]. Six of the included articles (67%) targeted medications – a moderate to high level of attention [41,45,46,47,48,49]. Other needs rising in priority are accidental self-harm, mobility and falls, behaviour, and caregiver support [75]. General care transition needs of older adults include intimate, caring relationships and self-care [76]. Caregivers of PLWD have a crucial role during care transitions, assisting with medication management, mobility (e.g. transportation), and preventing or easing psychological distress [59]. The need for clear and accurate information becomes more pressing, where PLWD and their caregivers need guidance to manage dementia-related risks like memory, mobility and falls [75,76,77]. Therefore, information is perhaps the dominant priority, as lapses in communication can lead to discontinuity of care, adverse events, and additional stress for PLWD and caregivers [59]. Five of the nine articles (56%) addressed information, suggesting room for improvement in how extensively the need is being addressed [41,44,45,47,49].

As a cost-effective and skilled workforce, volunteers can alleviate pressures on the healthcare system by supporting patients, staff, and trusts [28,41,42,43,49,78,79]. Involving volunteers in care transitions can help reduce emergency department/hospital admissions and system usage since health outcomes, such as the length of episodes and nutrition and hydration, are improved [41,43,48,49]. A robust approach to supporting community-based transitional care would integrate the voluntary sector with health and social structures [41,43,45,46,47,49]. When developing and implementing volunteer-led or supported interventions, adequate planning, training, volunteer engagement, resources, and consideration of users’ needs and priorities [44,49] must be thoroughly addressed. Incorporating discharge planning and goal setting as intervention components may help meet user needs and provide targeted support [46,47]. Ultimately, the promising findings from the articles suggest that volunteers are valuable resources and can effectively facilitate interventions that improve care transitions [41,42,43,46,48,49]. Another critical consideration for volunteers contributing to the healthcare workforce is the sustainability and scalability of their role. At the level of the volunteer, previous research has acknowledged that providing ongoing support for unknown situations, working with a well-established volunteer community organization, and having a designated volunteer coordinator are successful determinants of introducing and maintaining volunteer programming [80].

These findings are meaningful for stakeholders, including hospital administrators and management focused on resource efficiency, healthcare providers aiming to sustain progress, case managers and discharge planners, and system-level decision makers developing and implementing the interventions. Considering research from various regions, we believe this approach can be generalized and relevant for addressing a global challenge like dementia. Volunteer support is suggested to improve the quality of care transitions across varying systems and settings. Leveraging volunteers to support PLWD provides practical support to ensure smoother movement from hospital to home. This improves service integration, reduces disruptions, and addresses the specific needs of PLWD.

### Future Research

Since none of the papers explicitly describe *how* the volunteer-led/supported care transition interventions involved PLWD, this indicates a significant gap in evidence. Future research would benefit from a greater understanding of how volunteer-based integrated care models could be used as a sustainable strategy, leveraging the skills and knowledge of trained volunteers. Destabilization from hospital visits may cause the needs of PLWD to change or reprioritize, warranting further research to identify and evaluate transitional care needs. An adapted CANE instrument with updated items, co-designed with PLWD and their supports, would be valuable in mapping what should be targeted by volunteer-engaged initiatives. Future research is also needed to understand the impact of care transitions facilitated by volunteers on the caregivers of PLWD. Last, interventions engaging volunteers could be closely developed with the health and social care system to offer respite care and target caregiver outcomes, including burden, anxiety, and depression.

### Limitations

While this secondary analysis aimed to provide a focused review, several limitations must be acknowledged. The authors acknowledge the limitation of not involving the lived experience of dementia representation in the research team’s analysis and interpretation. In turn, researchers must intentionally and thoughtfully engage PLWD and their caregivers to understand the PLWD considerations for volunteer-led/involved transitional care support. The initial scoping review involved both academic and grey literature. Some articles, particularly non-empirical research articles, were not meant for a scoping review and needed more comprehensive information regarding the interventions and their impacts on patient outcomes, creating challenges in assessing the effectiveness of the interventions or whether they were tested on the included samples. Furthermore, 2024 and non-English literature articles are excluded from the scoping review, potentially excluding up-to-date or culturally specific insights on volunteer-led/supported care transition interventions.

The secondary analysis articles from the scoping review were selected using only one screener (SB). However, all the articles were reviewed by the senior author (MS). The varied level of detail across the articles, especially those that were a protocol, feasibility and acceptability study, training curriculum, or guide rather than impact assessments, complicated the data extraction process. In some instances, the inclusion or exclusion of PLWD or PWCI had to be inferred from the context. It cannot be assumed that all levels of cognitive impairment or stages of dementia were included. Generally, studies may exclude severe cases impacting intervention participation, such as difficulties with informed consent, capacity, and language and communication [81]. One article in the secondary analysis excluded those at risk of mortality, a common circumstance with advanced dementia [46,82]. Another based participation on MMSE score, excluding those with moderate to severe cognitive impairment (MMSE ≤ 20) [49].

## Conclusion

Volunteer-led/supported interventions that facilitate transitions from hospital to home positively impact the experience, health, and functioning of PLWD. While the interventions addressed many of their needs, the foremost needs of community-dwelling PLWD were not the primary focus. Prioritizing the less critical needs indicates that a more targeted approach is necessary. Flexible, personalized care strategies for vulnerable populations must consider all aspects of a patient’s condition—physical, cognitive, and emotional. Optimal care delivery can be achieved through intersectoral collaboration between healthcare and social systems.

## Additional Files

The additional files for this article can be found as follows:

10.5334/ijic.9056.s1 Supplemental Table 1. Scoping Review Search.

10.5334/ijic.9056.s2 Supplemental Table 2. Results.

10.5334/ijic.9056.s3 Supplemental Table 3. Quality Assessment.

## References

[1] Kanasi E, Ayilavarapu S, Jones J. The aging population: demographics and the biology of aging. Periodontology 2000. 2016;72:13–8. DOI: 10.1111/prd.12126 27501488

[2] Cao Q, Tan C-C, Xu W, Hu H, Cao X-P, Dong Q, et al. The Prevalence of Dementia: A Systematic Review and Meta-Analysis. JAD. 2020;73:1157–66. DOI: 10.3233/JAD-191092 31884487

[3] Lisko I, Kulmala J, Annetorp M, Ngandu T, Mangialasche F, Kivipelto M. How can dementia and disability be prevented in older adults: where are we today and where are we going? J Intern Med. 2021;289:807–30. DOI: 10.1111/joim.13227 33314384 PMC8248434

[4] Cadieux M-A, Garcia LJ, Patrick J. Needs of People With Dementia in Long-Term Care: A Systematic Review. Am J Alzheimers Dis Other Demen. 2013;28:723–33. DOI: 10.1177/1533317513500840 24005852 PMC10852926

[5] Hancock GA, Woods B, Challis D, Orrell M. The needs of older people with dementia in residential care. Int J Geriat Psychiatry. 2006;21:43–9. DOI: 10.1002/gps.1421 16323258

[6] Black BS, Johnston D, Leoutsakos J, Reuland M, Kelly J, Amjad H, et al. Unmet needs in community-living persons with dementia are common, often non-medical and related to patient and caregiver characteristics. Int Psychogeriatr. 2019;31:1643–54. DOI: 10.1017/S1041610218002296 30714564 PMC6679825

[7] Bossen AL, Pringle Specht JK, McKenzie SE. Needs of People with Early-Stage Alzheimer’s Disease: Reviewing the Evidence. J Gerontol Nurs. 2009;35:8–15. DOI: 10.3928/00989134-20090301-01 19326824

[8] Von Kutzleben M, Schmid W, Halek M, Holle B, Bartholomeyczik S. Community-dwelling persons with dementia: What do they need? What do they demand? What do they do? A systematic review on the subjective experiences of persons with dementia. Aging & Mental Health. 2012;16:378–90. DOI: 10.1080/13607863.2011.614594 22250961

[9] Mazurek J, Szcześniak D, Urbańska K, Dröes R-M, Rymaszewska J. Met and unmet care needs of older people with dementia living at home: Personal and informal carers’ perspectives. Dementia. 2019;18:1963–75. DOI: 10.1177/1471301217733233 28958171

[10] Brown M, Tolson D, Ritchie L. Changing needs in advanced dementia. Nursing Older People. 2020;32:14–20. DOI: 10.7748/nop.2020.e1204 32431132

[11] Bergman H, Borson S, Jessen F, Krolak-Salmon P, Pirani A, Rasmussen J, et al. Dementia and comorbidities in primary care: a scoping review. BMC Prim Care. 2023;24:277. DOI: 10.1186/s12875-023-02229-9 38097969 PMC10720181

[12] Motzek T, Werblow A, Tesch F, Marquardt G, Schmitt J. Determinants of hospitalization and length of stay among people with dementia – An analysis of statutory health insurance claims data. Archives of Gerontology and Geriatrics. 2018;76:227–33. DOI: 10.1016/j.archger.2018.02.015 29573708

[13] Resnick B, Boltz M, Galik E, Kuzmik A, McPherson R, Drazich B, et al. Measurement of Physical Activity Among Hospitalized Older Adults Living With Dementia. Rehabil Nurs. 2024;49:115–24. DOI: 10.1097/RNJ.0000000000000464 38904657 PMC11222057

[14] Kane RL, Ouslander JG. Dementia as a Moving Target. J American Geriatrics Society. 2012;60:980–2. DOI: 10.1111/j.1532-5415.2012.03918.x 22587858

[15] Pritchard E, Cussen A, Delafosse V, Swift M, Jolliffe L, Yeates H. Interventions supporting caregiver readiness when caring for patients with dementia following discharge home: A mixed-methods systematic review. Australas J Ageing. 2020;39. DOI: 10.1111/ajag.12765 31944506

[16] Knox S, Downer B, Haas A, Ottenbacher KJ. Successful Discharge to Community From Home Health Less Likely for People in Late Stages of Dementia. Journal of Geriatric Physical Therapy. 2023. DOI: 10.1519/JPT.0000000000000383 PMC10990837 38133896

[17] Saragosa M, Kuluski K, Okrainec K, Jeffs L. “Seeing the day-to-day situation”: A grounded theory of how persons living with dementia and their family caregivers experience the hospital to home transition and beyond. Journal of Aging Studies. 2023;65:101132. DOI: 10.1016/j.jaging.2023.101132 37268377

[18] Cohen CA, Pushkar D. Transitions in Care: Lessons Learned From a Longitudinal Study of Dementia Care. The American Journal of Geriatric Psychiatry. 1999;7:139–46. DOI: 10.1097/00019442-199905000-00007 10322241

[19] Allen J, Woolford M, Livingston PM, Lobchuk M, Muldowney A, Hutchinson AM. Informal carer support needs, facilitators and barriers in transitional care for older adults from hospital to home: A scoping review. Journal of Clinical Nursing. 2023;32:6773–95. DOI: 10.1111/jocn.16767 37272211

[20] *A step toward understanding health care trajectories of people living with dementia*. Ottawa, Ontario: Canadian Institute for Health Information; 2024.

[21] Leithaus M, Beaulen A, de Vries E, Goderis G, Flamaing J, Verbeek H, et al. Integrated Care Components in Transitional Care Models from Hospital to Home for Frail Older Adults: A Systematic Review. Int J Integr Care. n.d.;22:28. DOI: 10.5334/ijic.6447 PMC9248982 35855092

[22] Petersen HV, Foged S, Nørholm V. “It is two worlds” cross-sectoral nurse collaboration related to care transitions: A qualitative study. Journal of Clinical Nursing. 2019;28:1999–2008. DOI: 10.1111/jocn.14805 30706557

[23] O’Donnell R, Savaglio M, Skouteris H, Banaszak-Holl J, Moranl C, Morris H, et al. The Effectiveness of Transition Interventions to Support Older Patients From Hospital to Home: A Systematic Scoping Review. J Appl Gerontol. 2021;40:1628–36. DOI: 10.1177/0733464820968712 33155499

[24] Liebzeit D, Rutkowski R, Arbaje AI, Fields B, Werner NE. A scoping review of interventions for older adults transitioning from hospital to home. Journal of the American Geriatrics Society. 2021;69:2950–62. DOI: 10.1111/jgs.17323 34145906 PMC8497409

[25] Carpenter J, Spencer B, Moreira Da Souza T, Cho Y, Brett J. Exploring lessons from Covid-19 for the role of the voluntary sector in integrated care systems. Health Social Care Comm. 2022;30. DOI: 10.1111/hsc.14062 PMC9874554 36190115

[26] Nelson MLA, Saragosa M, Singh H, Yi J. Examining the Role of Third Sector Organization Volunteers in Facilitating Hospital-to-Home Transitions for Older Adults – a Collective Case Study. Int J Integr Care n.d.;24:16. DOI: 10.5334/ijic.7670 PMC10906339 38434712

[27] Sandhu S, Xu J, Blanchard L, Eisenson H, Crowder C, Munoz VS, et al. A Community Resource Navigator Model: Utilizing Student Volunteers to Integrate Health and Social Care in a Community Health Center Setting. International Journal of Integrated Care. 2021;21:2. DOI: 10.5334/ijic.5501 PMC7863845 33597833

[28] Fields NL, Roark EM, Xu L. Leveraging volunteers to support dementia family caregivers: An innovative approach to care and support. Bridging the Family Care Gap, Elsevier; 2021, p. 387–405. DOI: 10.1016/B978-0-12-813898-4.00013-0

[29] Lourens GM, Daniels-Felix DK. Hospital volunteerism as human resource solution: Motivation for both volunteers and the public health sector. SA j Hum Resour Manag. 2017;1. DOI: 10.4102/sajhrm.v15i0.813

[30] Goehner A, Kricheldorff C, Bitzer EM. Trained volunteers to support chronically ill, multimorbid elderly between hospital and domesticity – a systematic review of one-on-one-intervention types, effects, and underlying training concepts. BMC Geriatr. 2019;19:126. DOI: 10.1186/s12877-019-1130-2 31046693 PMC6498473

[31] Nelson MLA, Saragosa M, Singh H, Yi J. Examining the Role of Third Sector Organization Volunteers in Facilitating Hospital-to-Home Transitions for Older Adults – a Collective Case Study. International Journal of Integrated Care. 2024;24:16. DOI: 10.5334/ijic.7670 PMC10906339 38434712

[32] Lai FTT, Wong EL, Tam ZP, Cheung AW, Lau M-C, Wu C-M, et al. Association of volunteer-administered home care with reduced emergency room visits and hospitalization among older adults with chronic conditions: A propensity-score-matched cohort study. International Journal of Nursing Studies. 2022;127:104158. DOI: 10.1016/j.ijnurstu.2021.104158 35092873

[33] Law YW, Lok RHT, Chiang B, Lai CCS, Tsui SHM, Chung PYJ, et al. Effects of Community-Based Caring Contact in Reducing Thwarted Belongingness Among Postdischarge Young Adults With Self-Harm: Randomized Controlled Trial. JMIR Form Res. 2023;7:e43526. DOI: 10.2196/43526 37585260 PMC10468708

[34] Nelson M, MacEachern E, Saragosa M, Jhaji S, Thombs R, Thom S, et al. Synthesizing evidence regarding hospital to home transitions supported by volunteers of third sector organization: A scoping review [Under review]. Health Soc Care Community; n.d.

[35] Ruggiano N, Perry TE. Conducting secondary analysis of qualitative data: Should we, can we, and how? Qualitative Social Work. 2019;18:81–97. DOI: 10.1177/1473325017700701 30906228 PMC6428200

[36] Long-Sutehall T, Sque M, Addington-Hall J. Secondary analysis of qualitative data: a valuable method for exploring sensitive issues with an elusive population? Journal of Research in Nursing. 2011;16:335–44. DOI: 10.1177/1744987110381553

[37] Reynolds T, Thornicroft G, Abas M, Woods B, Hoe J, Leese M, et al. Camberwell Assessment of Need for the Elderly (CANE): Development, validity and reliability. Br J Psychiatry. 2000;176:444–52. DOI: 10.1192/bjp.176.5.444 10912220

[38] Van Der Roest HG, Meiland FJM, Comijs HC, Derksen E, Jansen APD, Van Hout HPJ, et al. What do community-dwelling people with dementia need? A survey of those who are known to care and welfare services. Int Psychogeriatr. 2009;21:949. DOI: 10.1017/S1041610209990147 19602305

[39] Van Der Ploeg ES, Bax D, Boorsma M, Nijpels G, Van Hout HP. A cross-sectional study to compare care needs of individuals with and without dementia in residential homes in the Netherlands. BMC Geriatr. 2013;13:51. DOI: 10.1186/1471-2318-13-51 23706150 PMC3691835

[40] Curnow E, Rush R, Maciver D, Górska S, Forsyth K. Exploring the needs of people with dementia living at home reported by people with dementia and informal caregivers: a systematic review and Meta-analysis. Aging & Mental Health. 2021;25:397–407. DOI: 10.1080/13607863.2019.1695741 31791140

[41] Dye C, Willoughby D, Aybar-Damali B, Grady C, Oran R, Knudson A. Improving Chronic Disease Self-Management by Older Home Health Patients through Community Health Coaching. IJERPH. 2018;15:660. DOI: 10.3390/ijerph15040660 29614803 PMC5923702

[42] Nesta. Helping in hospitals: A guide to high impact volunteering in hospitals; 2016.

[43] Elston J, Gradinger F, Asthana S, Fox M, Dawson L, Butler D, et al. Impact of ‘Enhanced’ Intermediate Care Integrating Acute, Primary and Community Care and the Voluntary Sector in Torbay and South Devon, UK. International Journal of Integrated Care. 2022;22:14. DOI: 10.5334/ijic.5665 PMC8855731 35282155

[44] McLeod E, Bywaters P, Tanner D, Hirsch M. For the Sake of their Health: Older Service Users’ Requirements for Social Care to Facilitate Access to Social Networks Following Hospital Discharge. British Journal of Social Work. 2008;38:73–90. DOI: 10.1093/bjsw/bcl341

[45] Eaton AD, Carusone SC, Ceranto A, Craig SL, Busch A, McCullagh JW. Training Peers to Ease Hospital Discharge: A Community–Clinical Partnership in Complex HIV Care. Progress in Community Health Partnerships. 2021;15:225–60. DOI: 10.1353/cpr.2021.0024 34248066

[46] Eaton AD, Chan Carusone S, Craig SL, Telegdi E, McCullagh JW, McClure D, et al. The ART of conversation: feasibility and acceptability of a pilot peer intervention to help transition complex HIV-positive people from hospital to community. BMJ Open. 2019;9:e026674. DOI: 10.1136/bmjopen-2018-026674 PMC6475144 30928956

[47] Deforge BR, Belcher JR. The Longitudinal Discharge Planning and Treatment Model (LDPTM): Part 2. Social Work in Mental Health. 2005;3:33–61. DOI: 10.1300/J200v03n04_03

[48] Hung D, Truong Q, Yakir M, Nicosia F. Hospital-Community Partnerships to Aid Transitions for Older Adults: Applying the Care Transitions Framework. Journal of Nursing Care Quality. 2018;33:221–8. DOI: 10.1097/NCQ.0000000000000294 29035905

[49] Wong FK, Ho MM, Yeung S, Tam SK, Chow SK. Effects of a health-social partnership transitional program on hospital readmission: A randomized controlled trial. Social Science & Medicine. 2011;73:960–9. DOI: 10.1016/j.socscimed.2011.06.036 21839564

[50] Yous M-L, Coker E, Hunter PV, Fisher KA, Sue JL, Nicula M, et al. Acceptability and preliminary effects of the volunteer-supported Meaningful Moments program to engage older adults with advanced dementia on a hospital-based specialized dementia care unit: a mixed methods study. BMC Geriatr. 2024;24:593. DOI: 10.1186/s12877-024-05194-9 38992599 PMC11238390

[51] Hall CL, Brooke J, Pendlebury ST, Jackson D. What is the Impact of Volunteers Providing Care and Support for People with Dementia in Acute Hospitals? A Systematic Review. Dementia. 2019;18:1410–26. DOI: 10.1177/1471301217713325 28587482

[52] Kessler D, Egan M, Kubina L-A. Peer support for stroke survivors: a case study. BMC Health Serv Res. 2014;14:256. DOI: 10.1186/1472-6963-14-256 24935460 PMC4070336

[53] Buys DR, Campbell AD, Godfryd A, Flood K, Kitchin E, Kilgore ML, et al. Meals Enhancing Nutrition After Discharge: Findings from a Pilot Randomized Controlled Trial. Journal of the Academy of Nutrition and Dietetics. 2017;117:599–608. DOI: 10.1016/j.jand.2016.11.005 28065635 PMC5368006

[54] Reynolds W, Lauder W, Sharkey S, Maciver S, Veitch T, Cameron D. The effects of a transitional discharge model for psychiatric patients. Psychiatric Ment Health Nurs. 2004;11:82–8. DOI: 10.1111/j.1365-2850.2004.00692.x 14723643

[55] Banovic S, Zunic L, Sinanovic O. Communication Difficulties as a Result of Dementia. Mater Sociomed. 2018;30:221. DOI: 10.5455/msm.2018.30.221-224 30515063 PMC6195406

[56] Piraino E, Heckman GA, Glenny C, Stolee P. Transitional care programs: who is left behind? A systematic review. Int J Integr Care. 2012;12. DOI: 10.5334/ijic.805 PMC3601531 23593046

[57] Cheng S-T. Dementia Caregiver Burden: a Research Update and Critical Analysis. Curr Psychiatry Rep. 2017;19:64. DOI: 10.1007/s11920-017-0818-2 28795386 PMC5550537

[58] Connors MH, Seeher K, Teixeira-Pinto A, Woodward M, Ames D, Brodaty H. Dementia and caregiver burden: A three-year longitudinal study. Int J Geriat Psychiatry. 2020;35:250–8. DOI: 10.1002/gps.5244 31821606

[59] Kable A, Chenoweth L, Pond D, Hullick C. Health professional perspectives on systems failures in transitional care for patients with dementia and their carers: a qualitative descriptive study. BMC Health Serv Res. 2015;15:567. DOI: 10.1186/s12913-015-1227-z 26684210 PMC4683856

[60] Lethin C, Renom-Guiteras A, Zwakhalen S, Soto-Martin M, Saks K, Zabalegui A, et al. Psychological well-being over time among informal caregivers caring for persons with dementia living at home. Aging & Mental Health. 2017;21:1138–46. DOI: 10.1080/13607863.2016.1211621 27463390

[61] Gorenko JA, Moran C, Flynn M, Dobson K, Konnert C. Social Isolation and Psychological Distress Among Older Adults Related to COVID-19: A Narrative Review of Remotely-Delivered Interventions and Recommendations. J Appl Gerontol. 2021;40:3–13. DOI: 10.1177/0733464820958550 32914668

[62] Haase KR, Cosco T, Kervin L, Riadi I, O’Connell ME. Older Adults’ Experiences With Using Technology for Socialization During the COVID-19 Pandemic: Cross-sectional Survey Study. JMIR Aging. 2021;4:e28010. DOI: 10.2196/28010 33739929 PMC8074950

[63] Sin F, Berger S, Kim I-J, Yoon D. Digital Social Interaction in Older Adults During the COVID-19 Pandemic. Proc ACM Hum-Comput Interact. 2021;5:1–20. DOI: 10.1145/347952436644216

[64] Lauriks S, Reinersmann A, Van Der Roest HG, Meiland F, Davies R, Moelaert F, et al. Review of ICT-Based Services for Identified Unmet Needs in People with Dementia. In: Mulvenna MD, Nugent CD, editors. Supporting People with Dementia Using Pervasive Health Technologies, London: Springer London; 2010, p. 37–61. DOI: 10.1007/978-1-84882-551-2_4

[65] Chen K, Lou VWQ, Lo SSC. A Tablet-Based Volunteer-Mediated Intervention for Cognitively Impaired Older People: A Pretest–Posttest. Research on Social Work Practice. 2020;30:288–97. DOI: 10.1177/1049731519863103

[66] Morgan B, Stites SD, Greenfield F, Fisher L, Kalafsky M, Hodgson N, et al. Time out weekly smile: A pilot test of a virtual respite program. Geriatric Nursing. 2023;54:178–83. DOI: 10.1016/j.gerinurse.2023.09.003 37797545 PMC10955550

[67] Rosebush CE, Stabler H, Nkimbeng M, Louwagie K, Fields NL, Jutkowitz E, et al. The Porchlight Project: A Pilot Study to Adapt the Senior Companion Program to Enhance Memory Care Services and Supports. Gerontology and Geriatric Medicine. 2021;7:233372142110176. DOI: 10.1177/23337214211017651 PMC8127785 34036120

[68] Sun W, Bartfay E, Smye V, Biswas S, Newton D, Pepin M, et al. Living well with dementia: The role volunteer-based social recreational programs in promoting social connectedness of people with dementia and their caregivers. Aging & Mental Health. 2022;26:1949–62. DOI: 10.1080/13607863.2021.1950614 34353187

[69] Xu L, Fields NL, Daniel KM, Cipher DJ, Troutman BA. Reminiscence and Digital Storytelling to Improve the Social and Emotional Well-Being of Older Adults With Alzheimer’s Disease and Related Dementias: Protocol for a Mixed Methods Study Design and a Randomized Controlled Trial. JMIR Res Protoc. 2023;12:e49752. DOI: 10.2196/49752 37676706 PMC10514775

[70] Gisch UA, Ahlers E, Lee D, Heuser-Collier I, Somasundaram R. A program for volunteers accompanying older patients with cognitive dysfunction to improve the quality of emergency department care: A pilot study. Geriatric Nursing. 2022;48:94–102. DOI: 10.1016/j.gerinurse.2022.09.001 36155315

[71] Miranda-Castillo C, Woods B, Orrell M. The needs of people with dementia living at home from user, caregiver and professional perspectives: a cross-sectional survey. BMC Health Serv Res. 2013;13:43. DOI: 10.1186/1472-6963-13-43 23379786 PMC3568411

[72] Kaasalainen S, Dolovich L, Papaioannou A, Holbrook A, Lau E, Ploeg J, et al. The process of medication management for older adults with dementia: Medication management in dementia. Journal of Nursing and Healthcare of Chronic Illness. 2011;3:407–18. DOI: 10.1111/j.1752-9824.2011.01114.x

[73] Knox S, Downer B, Haas A, Middleton A, Ottenbacher KJ. Function and Caregiver Support Associated With Readmissions During Home Health for Individuals With Dementia. Archives of Physical Medicine and Rehabilitation. 2020;101:1009–16. DOI: 10.1016/j.apmr.2019.12.021 32035139 PMC7279123

[74] Stockwell-Smith G, Moyle W, Marshall AP, Argo A, Brown L, Howe S, et al. Hospital discharge processes involving older adults living with dementia: An integrated literature review. Journal of Clinical Nursing. 2018;27. DOI: 10.1111/jocn.14144 29076202

[75] Toles M, Leeman J, McKay MH, Covington J, Hanson LC. Adapting the Connect-Home transitional care intervention for the unique needs of people with dementia and their caregivers: A feasibility study. Geriatric Nursing. 2022;48:197–202. DOI: 10.1016/j.gerinurse.2022.09.016 36274509 PMC9749405

[76] Allen J, Hutchinson AM, Brown R, Livingston PM. User experience and care for older people transitioning from hospital to home: Patients’ and carers’ perspectives. Health Expectations. 2018;21:518–27. DOI: 10.1111/hex.12646 29120529 PMC5867324

[77] Moore JR, Sullivan MM. Enhancing the ADMIT Me Tool for Care Transitions for Individuals With Alzheimer’s Disease. J Gerontol Nurs. 2017;43:32–8. DOI: 10.3928/00989134-20170112-01 28095582

[78] Handy F, Brudney J. When to Use Volunteer Labor Resources? An Organizational Analysis for Nonprofit Management. Vrijwillige Inzet Onderzoch. 2007;4:91–100.

[79] Jago L, Deery M. The role of human resource practices in achieving quality enhancement and cost reduction: an investigation of volunteer use in tourism organisations. Int J Contemp Hospitality Mngt. 2002;14:229–36. DOI: 10.1108/09596110210433754

[80] Fredriksen E, Martinez S, Moe CE, Thygesen E. Key challenges and best practices in the coordination of volunteers in healthcare services: A qualitative systematic review. Health & Social Care in the Community. 2021;29:1607–20. DOI: 10.1111/hsc.13261 33368759

[81] Motta-Ochoa R, Bresba P, Da Silva Castanheira J, Lai Kwan C, Shaffer S, Julien O, et al. “When I hear my language, I travel back in time and I feel at home”: Intersections of culture with social inclusion and exclusion of persons with dementia and their caregivers. Transcult Psychiatry. 2021;58:828–43. DOI: 10.1177/13634615211001707 33957816 PMC8637382

[82] Mitchell SL, Teno JM, Kiely DK, Shaffer ML, Jones RN, Prigerson HG, et al. The Clinical Course of Advanced Dementia. N Engl J Med. 2009;361:1529–38. DOI: 10.1056/NEJMoa0902234. 19828530 PMC2778850

