# Supplementary material for: Volunteer-supported Care Transition Interventions for People Living with Dementia: A Secondary Analysis of a Scoping Review
Source: Int J Integr Care. 2025 May 21;25(2):16. doi: 10.5334/ijic.9056 (PMC12101112; doi:10.5334/ijic.9056)
Supplement: Supplemental Table 1. — Scoping Review Search. [file ijic-25-2-9056-s1.pdf]

Appendix A. Scoping Review Database search strategies

Database search strategies.

1. Scopus

((TITLE-ABS-KEY ((voluntary OR unpaid OR un-paid OR uncompensated OR un-compensated OR informal OR nongovernmental OR non-governmental OR ngo OR non-profit OR nonprofit OR "non profit" OR "not for profit" OR not-for-profit) W/3 (work* OR care* OR service* OR support* OR involvement OR health* OR hospice* OR help* OR counsel* OR staff OR personnel OR provider* OR group* OR organization* OR association * OR agenc* OR network* OR sector* OR program*))) OR (TITLE-ABS-KEY (volunteer*))) AND ((TITLE-ABS-KEY (discharg* W/4 (patient* OR home* OR house* OR residence* OR community OR hospital* OR er))) OR (TITLE-ABS-KEY (post-discharg* OR "post discharg*" OR aftercare OR after-care OR "transition* care" OR "follow up care" OR "follow-up care" OR "follow-up-care" OR post-hospital OR "post hospital" OR "after hospital" OR after-hospital OR "self care")) OR (TITLE-ABS-KEY (hospital W/4 (home OR house OR community OR residence*))) OR (TITLE-ABS-KEY (transition* W/4 (home OR house OR community OR residence*))) OR (TITLE-ABS-KEY (community W/4 (integrat* OR reintegrat* OR re-integrat* OR reentry OR re-entry OR reentrance OR re-entrance))))

2. Cochrane

#1 MeSH descriptor: [Volunteers] this term only
#2 MeSH descriptor: [Voluntary Health Agencies] explode all trees
#3 (volunteer*):ti,ab,kw in Cochrane Reviews, Cochrane Protocols
#4 (voluntary or unpaid or "un-paid" or uncompensated or "un-compensated" or informal or nongovernmental or "non-governmental" or NGO or "non-profit" or nonprofit or "non profit" or "not for profit" or "not-for-profit"):ti,ab,kw in Cochrane Reviews, Cochrane Protocols
#5 MeSH descriptor: [Hospital Auxiliaries] explode all trees
#6 MeSH descriptor: [Charities] this term only
#7 MeSH descriptor: [Faith-Based Organizations] this term only
#8 MeSH descriptor: [Self-Help Groups] explode all trees
#9 MeSH descriptor: [Community Health Services] this term only
#10 MeSH descriptor: [Adult Day Care Centers] this term only
#11 MeSH descriptor: [Senior Centers] this term only
#12 MeSH descriptor: [Community Networks] explode all trees
#13 {OR #1-#12} in Cochrane Reviews, Cochrane Protocols
#14 MeSH descriptor: [Patient Discharge] explode all trees
#15 (post-discharge* or "post discharge*"):ti,ab,kw in Cochrane Reviews, Cochrane Protocols
#16 (discharg* NEAR/4 (home* or house* or residence* or community or hospital* or ER)):ti,ab,kw in Cochrane Reviews, Cochrane Protocols
#17 (hospital NEAR/4 (home or house or community or residence*)):ti,ab,kw in Cochrane Reviews, Cochrane Protocols
#18 (transition NEAR/4 (home or house or community or residence*)):ti,ab,kw in Cochrane Reviews, Cochrane Protocols
#19 MeSH descriptor: [Self Care] explode all trees
#20 MeSH descriptor: [Aftercare] this term only
#21 (aftercare or after-care or "transition care" or transition-care):ti,ab,kw in Cochrane Reviews, Cochrane Protocols
#22 (post-hospital* or posthospital* or after-hospital or "after hospital"):ti,ab,kw in Cochrane Reviews, Cochrane Protocols
#23 (communit* NEAR/4 (integrat* or reintegrat* or re-integrat* or reentry or re-entry or reentrance or re-entrance)):ti,ab,kw in Cochrane Reviews, Cochrane Protocols
#24 {OR #14-#23}
#25 #13 AND #24

3. Medline

1 Volunteers/
2 exp Voluntary Health Agencies/
3 volunteer*.tw,kf.
4 ((voluntary or unpaid or un-paid or uncompensated or un-compensated or informal or nongovernmental or non-governmental or NGO or non-profit or nonprofit or non profit or "not for profit" or not-for-profit) adj3 (work* or care* or service* or support* or involvement or health* or hospice* or help* or counsel* or staff or personnel or provider* or group* or organi#ation* or association* or agenc* or network* or sector* or program*)).tw,kf.
5 exp Hospital Auxiliaries/
6 Charities/
7 Faith-Based Organizations/
8 exp Self-Help Groups/
9 Community Health Services/
10 Adult Day Care Centers/
11 Senior Centers/
12 Community Networks/
13 or/1-12 [volunteer concept]
14 Patient Discharge/
15 (post-discharge* or post discharge*).tw,kf.
16 (discharg* adj4 (home* or house* or residence* or community or hospital* or ER)).tw,kf.
17 (hospital adj4 (home or house or community or residence*)).tw,kf.
18 (transition* adj4 (home or house or community or residence*)).tw,kf.
19 exp Self Care/
20 Aftercare/
21 (aftercare or after-care or transition care or transition-care).tw,kf.
22 (post-hospital* or posthospital* or after-hospital or after hospital).tw,kf.
23 (community adj4 (integrat* or reintegrat* or re-integrat* or reentry or re-entry or reentrance or re-entrance)).tw,kf.
24 or/14-23 [transition from hospital to home/community concept]
25 13 and 24 [Volunteer concept + transition from hospital to home/community concept]
26 Palliative Care/
27 exp Terminal Care/
28 Hospices/
29 or/26-28 [palliative care concept]
30 25 not 29 [to remove palliative studies]
31 30 not ((exp infant/ or exp child/ or exp adolescent/) not exp adult/) [to remove studies indexed as child only]

4. Joanna Briggs Institute

1 volunteer*.ti,hw.
2 ((voluntary or unpaid or un-paid or informal or nongovernmental or non-governmental or NGO or non-profit or nonprofit or non profit or "not for profit" or not-for-profit) adj3 (work* or care* or service* or support* or involvement or health* or hospice* or help* or counsel* or staff or personnel or provider* or group* or organi#ation* or association* or agenc* or network* or sector* or program*)).ti,hw.
3 (charity or charities).ti,hw.
4 ((self-help or self help or selfhelp or support) adj3 group*).ti,hw.
5 (communit* adj3 network*).ti,hw.
6 or/1-5 [volunteer concept]
7 (post-discharge* or post discharge*).ti,hw.
8 (discharg* adj4 (home* or house* or residence* or community or hospital* or ER)).ti,hw.
9 (hospital adj4 (home or house or community or residence*)).ti,hw.
10 (transition* adj4 (home or house or community or residence*)).ti,hw.
11 (aftercare or after-care or transition care or transition-care).ti,hw.
12 (post-hospital* or posthospital* or after-hospital or after hospital).ti,hw.
13 (community adj4 (integrat* or reintegrat* or re-integrat* or reentry or re-entry or reentrance or re-entrance)).ti,hw.
14 or/7-13 [transition from hospital to home/community concept]
15 6 and 14 [Volunteer concept + transition from hospital to home/community concept]

5. PsycInfo

1 volunteers/
2 exp prosocial behavior/
3 nonprofit organizations/
4 exp religious organizations/
5 exp support groups/
6 social support/
7 exp social networks/
8 social services/
9 community services/
10 home visiting programs/
11 independent living programs/
12 adult day care/
13 elder care/
14 aging in place/
15 home care/
16 home visiting programs/
17 volunteers.mh.
18 Voluntary Health Agencies.mh.
19 volunteer*.tw.
20 ((voluntary or unpaid or un-paid or uncompensated or un-compensated or informal or nongovernmental or non-governmental or NGO or non-profit or nonprofit or non profit or "not for profit" or not-for-profit) adj3 (work* or care* or service* or support* or involvement or health* or hospice* or help* or counsel* or staff or personnel or provider* or group* or organi#ation* or association* or agenc* or network* or sector* or program*)).tw.
21 or/1-20 [volunteer concept]
22 hospital discharge/
23 Patient Discharge.mh.
24 (post-discharge* or post discharge*).tw.
25 (discharg* adj4 (home* or house* or residence* or community or hospital* or ER)).tw.
26 (hospital adj4 (home or house or community or residence*)).tw.
27 (transition* adj4 (home or house or community or residence*)).tw.
28 aftercare/
29 aftercare.mh.
30 (aftercare or after-care or transition care or transition-care).tw.
31 (post-hospital* or posthospital* or after-hospital or after hospital).tw.
32 (community adj4 (integrat* or reintegrat* or re-integrat* or reentry or re-entry or reentrance or re-entrance)).tw.
33 or/22-32 [transition from hospital to home/community concept]
34 21 and 33 [Volunteer concept + transition from hospital to home/community concept]
35 palliative care/
36 terminally ill patients/
37 or/35-36 [palliative care concept]

38 34 not 37 [to remove palliative studies]
39 (dissertation abstract or book).pt.
40 38 not 39 [to remove dissertation and book publication types]

6. EMBASE

1 exp volunteer/
2 voluntary worker/
3 voluntary program/
4 ((voluntary or unpaid or un-paid or uncompensated or un-compensated or informal or nongovernmental or non-governmental or NGO or non-profit or nonprofit or non profit or "not for profit" or not-for-profit) adj3 (work* or care* or service* or support* or involvement or health* or hospice* or help* or counsel* or staff or personnel or provider* or group* or organi#ation* or association* or agenc* or network* or sector* or program*)).tw,kw.
5 volunteer*.tw,kw.
6 exp faith-based organization/
7 exp self help/
8 adult day care/
9 senior center/
10 community care/
11 exp community program/
12 exp non profit organization/
13 or/1-12 [volunteer concept]
14 hospital discharge/
15 aftercare/
16 self care/
17 community integration/
18 exp community reintegration/
19 (post-discharge* or post discharge*).tw,kw.
20 (discharg* adj3 (home* or house* or residence* or community or hospital* or ER)).tw,kw.
21 (hospital adj4 (home or house or community or residence*)).tw,kw.
22 (transition* adj4 (home or house or community or residence*)).tw,kw.
23 (aftercare or after-care or transition care or transition-care).tw,kw.
24 (post-hospital* or posthospital* or after-hospital or after hospital).tw,kw.
25 (community adj4 (integrat* or reintegrat* or re-integrat* or reentry or re-entry or reentrance or re-entrance)).tw,kw.
26 or/14-25 [transition from hospital to home/community concept]
27 13 and 26 [Volunteer concept + transition from hospital to home/community concept]
28 exp palliative therapy/
29 exp terminal care/
30 or/28-29 [palliative concept]
31 27 not 30 [to remove palliative studies]
32 31 not (exp juvenile/ not exp adult/) [to remove studies indexed as child only]

7. CINAHL

S1 (MH "Volunteer Workers")
S2 (MH "Volunteer Experiences")
S3 TI ((voluntary or unpaid or un-paid or uncompensated or un compensated or informal or nongovernmental or non-governmental or NGO or non-profit or nonprofit or non profit or "not for profit" or not-for-profit) N3 (work* or care* or service* or support* or involvement or health* or hospice* or help* or counsel* or staff or personnel or provider* or group* or organization* or association* or agenc* or network* or sector* or program*)) OR AB ((voluntary or unpaid or un paid or uncompensated or un-compensated or informal or nongovernmental or non governmental or NGO or non-profit or nonprofit or non profit or "not for profit" or not-for-profit) N3 (work* or care* or service* or support* or involvement or health* or hospice* or help* or counsel* or staff or personnel or provider* or group* or organization* or association* or agenc* or network* or sector* or program*))
S4 (MH "Charities")
S5 (MH "Faith-Based Organizations")
S6 (MH "Organizations, Nonprofit+")
S7 (MH "Support Groups")
S8 (MH "Uncompensated Care")
S9 (MH "Adult Day Center (Saba CCC)")
S10 (MH "Senior Centers")
S11 (MH "Community Networks")
S12 (MH "Community Health Services")
S13 S1 OR S2 OR S3 OR S4 OR S5 OR S6 OR S7 OR S8 OR S9 OR S10 OR S11 OR S12
S14 (MH "Patient Discharge+")
S15 TI (post-discharge* or post discharge*) OR AB (post-discharge* or post discharge*)
S16 TI ((discharg* N4 (home* or house* or residence* or community or hospital* or ER))) OR AB ((discharg* N4 (home* or house* or residence* or community or hospital* or ER)))
S17 TI ((hospital N4 (home or house or community or residence*))) OR AB ((hospital N4 (home or house or community or residence*)))
S18 TI (transition* N4 (home or house or community or residence*)) OR AB (transition* N4 (home or house or community or residence*))
S19 (MH "Self Care+")
S20 (MH "After Care")
S21 TI (aftercare or after-care or transition care or transition-care) OR AB (aftercare or after-care or transition care or transition-care)
S22 TI (post-hospital* or posthospital* or after hospital or after hospital) OR AB (post-hospital* or posthospital* or after hospital or after hospital)
S23 TI (community N4 (integrat* or reintegrat* or re-integrat* or reentry or re-entry or reentrance or re-entrance)) OR AB (community N4 (integrat* or reintegrat* or re integrat* or reentry or re entry or reentrance or re entrance))
S24 S14 OR S15 OR S16 OR S17 OR S18 OR S19 OR S20 OR S21 OR S22 OR S23
S25 S13 AND S24

8. Social Work Abstracts

1 volunteer*.mp.
2 ((voluntary or unpaid or un-paid or uncompensated or un-compensated or informal or nongovernmental or non-governmental or NGO or non-profit or nonprofit or non profit or "not for profit" or not-for-profit) adj3 (work* or care* or service* or support* or involvement or health* or hospice* or help* or counsel* or staff or personnel or provider* or group* or organi#ation* or association* or agenc* or network* or sector* or program*)).mp.
3 ((self-help or self help or support) adj2 group*).mp.
4 or/1-3 [volunteer concept]
5 (post-discharge* or post discharge*).mp.
6 (discharg* adj4 (home* or house* or residence* or community or hospital* or ER)).mp.
7 (hospital adj4 (home or house or community or residence*)).mp.
8 (transition* adj4 (home or house or community or residence*)).mp.
9 (aftercare or after-care or transition care or transition-care).mp.
10 (post-hospital* or posthospital* or after-hospital or after hospital).mp.
11 (community adj4 (integrat* or reintegrat* or re-integrat* or reentry or re-entry or reentrance or re-entrance)).mp.
12 or/5-11 [transition from hospital to home/community concept]
13 4 and 12 [Volunteer concept + transition from hospital to home/community concept]

9. Ageline

S1 AB volunteer* OR TI volunteer*
S2 AB ((voluntary or unpaid or un-paid or uncompensated or un compensated or informal or nongovernmental or non-governmental or NGO or non-profit or nonprofit or non profit or "not for profit" or not-for-profit) N3 (work* or care* or service* or support* or involvement or health* or help* or counsel* or staff or personnel or provider* or group* or organi#ation* or association* or agenc* or network* or sector* or program*)) OR TI ((voluntary or unpaid or un paid or uncompensated or un-compensated or informal or nongovernmental or non governmental or NGO or non-profit or nonprofit or non profit or "not for profit" or not-for-profit) N3 (work* or care* or service* or support* or involvement or health* or hospice* or help* or counsel* or staff or personnel or provider* or group* or organi#ation* or association* or agenc* or network* or sector* or program*))
S3 S1 OR S2
S4 AB (post-discharge* or "post discharge*" or aftercare or after-care or "transition care" or transition-care) OR TI (post-discharge* or "post discharge*" or aftercare or after-care or "transition care" or transition-care)
S5 AB (dicharg* N4 (home* or house* or residence* or community or hospital* or ER)) OR TI (dicharg* N4 (home* or house* or residence* or community or hospital* or ER))
S6 AB (discharg* N4 (home* or house* or residence* or community or hospital* or ER)) OR TI (discharg* N4 (home* or house* or residence* or community or hospital* or ER))
S7 AB (hospital N4 (home or house or community or residence*)) OR TI (hospital N4 (home or house or community or residence*))
S8 AB (transition* N4 (home or house or community or residence*)) OR TI (transition* N4 (home or house or community or residence*))
S9 AB (aftercare or after care or "transition care" or transition-care) OR TI (aftercare or after-care or "transition care" or transition-care)
S10 AB (post-hospital* or posthospital* or after hospital or "after hospital") OR TI (post-hospital* or posthospital* or after hospital or "after hospital")
S11 AB (community N4 (integrat* or reintegrat* or re-integrat* or reentry or re-entry or reentrance or re-entrance)) OR TI (community N4 (integrat* or reintegrat* or re
integrat* or reentry or re entry or reentrance or re entrance))
S12 S4 OR S5 OR S6 OR S7 OR S8 OR S9 OR S10 OR S11
S13 S3 AND S12

10. Sociological Abstracts

(MAINSUBJECT.EXACT.EXPLODE("Volunteers") OR (ab(voluntary OR unpaid OR un-paid OR informal OR nongovernmental OR non-governmental OR NGO OR non-profit OR nonprofit OR non profit OR "not for profit" OR not-for-profit) AND ab(work* OR care* OR service* OR support* OR involvement OR health* OR hospice* OR help* OR counsel* OR staff OR personnel OR provider* OR group* OR organi#ation* OR association* OR agenc* OR network* OR sector* OR program*)) OR (ti(voluntary OR unpaid OR un-paid OR informal OR nongovernmental OR non-governmental OR NGO OR non-profit OR nonprofit OR non profit OR "not for profit" OR not-for-profit) AND ti(work* OR care* OR service* OR support* OR involvement OR health* OR hospice* OR help* OR counsel* OR staff OR personnel OR provider* OR group* OR organi#ation* OR association* OR agenc* OR network* OR sector* OR program*)) OR (ti(volunteer*) OR ab(volunteer*))) AND (MAINSUBJECT.EXACT("Discharge") OR MAINSUBJECT.EXACT("After Care") OR (ab(post-discharge* OR post discharge*) OR ti(post-discharge* OR post discharge*)) OR (ab(discharg*) AND ab(home* OR house* OR residence* OR community OR hospital* OR ER)) OR (ti(discharg*) AND ti(home* OR house* OR residence* OR community OR hospital* OR ER)) OR (ti(hospital) AND ti(home OR house OR community OR residence*)) OR (ab(hospital) AND ab(home OR house OR community OR residence*)) OR (ab(transition*) AND ab(home OR house OR community OR residence*)) OR (ti(transition*) AND ti(home OR house OR community OR residence*)) OR (ti(aftercare OR after-care OR transition care OR transition-care) OR ti(aftercare OR after-care OR transition care OR transition-care)) OR (ab(aftercare OR after-care OR transition care OR transition-care) OR ab(aftercare OR after-care OR transition care OR transition-care)) OR (ab(post-hospital* OR posthospital* OR after-hospital OR after hospital) OR ab(post-hospital* OR posthospital* OR after-hospital OR after hospital)) OR (ti(post-hospital* OR posthospital* OR after-hospital OR after hospital) OR ti(post-hospital* OR posthospital* OR after-hospital OR after hospital)) OR (ti(community) AND ti(integrat* OR reintegrat* OR re-integrat* OR reentry OR re-entry OR reentrance OR re-entrance)) OR (ab(community) AND ab(integrat* OR reintegrat* OR re-integrat* OR reentry OR re-entry OR reentrance OR re-entrance)))
