# Supplementary material for: Volunteer-supported Care Transition Interventions for People Living with Dementia: A Secondary Analysis of a Scoping Review
Source: Int J Integr Care. 2025 May 21;25(2):16. doi: 10.5334/ijic.9056 (PMC12101112; doi:10.5334/ijic.9056)
Supplement: Supplemental Table 2. — Results. [file ijic-25-2-9056-s2.pdf]

Author/Year	Country	Study Design	Population	Nature of cognitive impairment	Intervention	Duration	Dosage	Setting	Intervention Deliverer	Patient Outcome Measures	Findings
Anonymous, 2016	United Kingdom	Comparison group designs or pre-post approach	Not specific	Dementia	Volunteer services including:<br>• Phone calls<br>• Home visits<br>• Mealtime assistance<br>• Dementia buddies	Varies by hospital/program	Varies by hospital/program	Hybrid (varies by hospital/program)	Volunteers, volunteer coordinator	• Patient experience, mood, and anxiety<br>• Nutrition and hydration levels<br>• Readmissions and length of stay<br>• Delay in transfer of care<br>• Number of falls	• Positive impacts on nutrition and hydration levels<br>• Positive impacts on releasing time to care<br>• Improvements on patient experience, mood, and anxiety<br>• No effects on readmissions, length of stay, delayed transfer of care, and number of falls
DeForge and Belcher, 2005	United States	Mixed methods protocol	Persons discharged from a psychiatric facility	Not specific	Inpatient and outpatient multidisciplinary teams engaged in:<br>• Discussion of client needs<br>• Discharge planning<br>• Community linkages	Not specified	Flexible depending on patient's needs	Not reported	Case manager, psychiatrist, nurse, social worker, psychologist, primary care health provider, vocational counselor, substance abuse counselor, consumer and family advocate, and volunteers	• Psychiatric symptoms and recurrent psychiatric episodes<br>• Quality of life, independence, and social and employment functioning<br>• Meeting basic needs<br>• Burden on the family	Not reported

Author/Year	Country	Study Design	Population	Nature of cognitive impairment	Intervention	Duration	Dosage	Setting	Intervention Deliverer	Patient Outcome Measures	Findings
Dye et al., 2018	United States	Non-randomized	Older adults (≥60 years) with a diagnosis of CHF, T2D, or CVDs	Alzheimer's, non-specific dementia, and non-specific cognitive impairment	Community members engaged in:<br>• Home visits<br>• Phone calls	4 months	Month 1: 3.5 hours/week (2 home visits and 3 phone calls)<br>Month 2: 3 hours/week (1 home visit and 4 phone calls)<br>Month 3: 2.5 hours/week (0 home visits and 4 phone calls)<br>Month 4: 2 hours/week (4 phone calls)	Hybrid	Volunteers, volunteer coordinator	• Hospital/ED readmission for CVD, T2D, or CHF<br>• Hospital/ED admission for fall, flu, or pneumonia<br>• Cost of admissions	Reduced hospital/ED admissions for CVD, T2D, CHF, falls, flu, or pneumonia
Eaton et al., 2019	Canada	Feasibility and acceptability evaluation of mixed methods study	HIV-positive patients discharged back to the community who actively use ilicit substances	HIV-associated neurocognitive disorder	Peer intervention involving:<br>• Pre-discharge goal setting<br>• Phone calls	7 weeks	1/day for the first 3 days then 1/week for the following 6 weeks	Remote	Volunteers	Not reported	•Intervention was feasible and acceptable for the target population<br>• Some barriers to phone call engagement
Eaton et al., 2021	Canada	Qualitative	Acutely hospitalized HIV-positive patients struggling with antiretroviral adherence and substance use	Not specific	Three-stage peer intervention involving:<br>• Volunteer matching with patients<br>• Pre-discharge goal setting<br>• Phone calls	7 weeks	1/day for the first 3 days then 1/week for the following 6 weeks	Remote	Inpatient nurse and volunteers	Not reported	• Success in validating patient feelings/experiences<br>• Success in tracking patient goals<br>• Difficulty in maintaining emotional connection over the phone

Author/Year	Country	Study Design	Population	Nature of cognitive impairment	Intervention	Duration	Dosage	Setting	Intervention Deliverer	Patient Outcome Measures	Findings
Elston et al., 2022	United Kingdom	Mixed methods	Older adults	Dementia	Multi-disciplinary teams engaged in:<br>• Discussing and planning patient care<br>• Providing proactive care<br>• Social prescribing<br>• Goal setting<br>• Coaching<br>• Providing practical and emotional support	12 weeks	Not reported	Not reported	General practitioners, pharmacists, voluntary sector well-being coordinators, community matrons, community nurses, occupational and physiotherapists, social workers, mental health liaison staff, health and social care coordinators found in other localities, and volunteers	• Average length of episode<br>• Bed-day rates<br>• ED admissions<br>• Proactive hospital discharges	• Reduced average length of episode and bed-day rates<br>• Reduced activity in the health and social care system
Hung et al., 2018	United States	Qualitative	Older adults (≥65 years)	Not specific	Community-based program involving:<br>• Discharge planning to implement teach-back methods<br>• Scheduling follow-up appointments<br>• Home visits<br>• Phone calls<br>• Providing community resources and services	7 months	Not reported	Hybrid	Health coaches and volunteers	• 30-day unplanned readmissions<br>• Meeting social needs<br>• Number/type of patient activations initiated<br>• Quality of life and satisfaction<br>• Readiness for care visits, communication with physicians, and understanding treatments and lifestyle changes<br>• Engagement with community resources	• Reduced 30-day unplanned readmissions<br>• Safety net and valuable transition program for patients<br>• Patient needs met

**Author/Year**	**Country**	**Study Design**	**Population**	**Nature of cognitive impairment**	**Intervention**	**Duration**	**Dosage**	**Setting**	**Intervention Deliverer**	**Patient Outcome Measures**	**Findings**
McLeod et al., 2008	United Kingdom	Qualitative	Older adults	Alzheimer's and non-specific cognitive impairment	Voluntary sector hospital aftercare social rehabilitation involving home visits	6-8 weeks	Approximately 1-1.5 hours/week	In-person	Volunteers and social care workers	Not reported	• Important for patient well-being to access services and social networks post discharge<br>• Older adults should not be discharged if too ill to cope<br>• Discharge services should be funded and coordinated<br>• Personal care needs should be integrated into extended intermediate care
Wong et al., 2011	Hong Kong	Randomized controlled trial	Older adults (≥60 years)	Not specific	Health-social partnership transitional care management program involving:<br>• Pre-discharge assessment<br>• Home visits<br>• Phone calls	4 weeks	Week 1: 1 home visit<br>Week 2: 1 phone call<br>Week 3: 1 home visit<br>Week 4: 1 phone call	Hybrid	Nurse case managers, trained volunteers, and social workers	• Hospital readmissions<br>• Quality of life<br>• Self-efficacy<br>• Satisfaction with care	• Reduced 28-day readmission<br>• Improved quality of life and self-efficacy<br>• Improved satisfaction with care
